# Posttransplant lymphoproliferative disease presenting as jejunal perforation

**DOI:** 10.4103/0971-4065.53330

**Published:** 2009-04

**Authors:** K. C. Krishna, G. Sivaramakrishna, P. M. Kumar, T. Kannan, K. M. Reddy, V. Sivakumar

**Affiliations:** Department of Nephrology, Sri Venkateswara Institute of Medical Sciences, Tirupati, India; 1 Department of Surgical Gastroenterology, Sri Venkateswara Institute of Medical Sciences, Tirupati, India; 2 Department of Medical Oncology, Sri Venkateswara Institute of Medical Sciences, Tirupati, India; 3 Department of Pathology, Sri Venkateswara Institute of Medical Sciences, Tirupati, India

**Figure 1:**
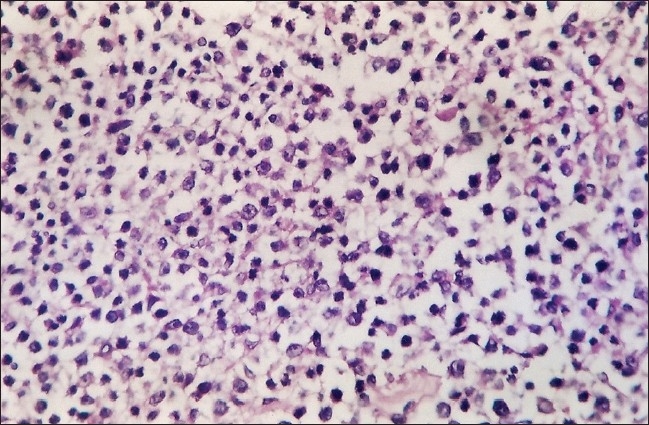
H and E, (10 × 10) jejunum showing diffuse large cell lymphomatous infiltration of all layers

Sir,

A forty-nine year-old male underwent live related renal transplantation thirteen years ago for end stage renal disease secondary to bilateral vesicoureteric reflux. He was on prednisolone 10 mg per day and azathioprine 125 mg per day for maintenance immunosuppression. He presented with pain in his abdomen and vomiting that had been prevalent since the past day. Evaluation was suggestive of hollow viscous perforation which was confirmed by computed tomography of the abdomen. As laparotomy revealed a perforation with growth in the jejunum, a jejunal segment resection was done followed by jejunojejunal anastamosis. Histopathological investigation revealed that the growth was a diffuse, large B cell lymphoma [Figure 1]. Immunohistochemistry confirmed it to be a B cell variant of diffuse large cell NonHodgkin's lymphoma of the small bowel. Viral serology showed significant Epstein barr virus (viral capsid antigen) IgM and Epstein barr virus (nuclear antigen) IgG levels being normal. The patient was maintained on CHOP (Cyclophosphamide, Adriamycin, Vincristine, and Prednisolone) regimen by a medical oncologist.

Renal transplant recipients carry a three to five fold higher risk of malignancy as compared to healthy people. Posttransplant lymphoproliferative disease (PTLD) is second only to skin cancers in contributing to 12% of posttransplant malignancies and its incidence increases with time.[1] The risk of developing PTLD is highest within the first year after the transplant, and the probability decreases thereafter.[2] The incidence increases with the age of the patient and the duration and cumulative dose of immunosuppressive therapy. It is associated with high morbidity and mortality. Cumulative doses and longer duration of systemic glucocorticoids and azathioprine increase the incidence of nonHodgkin's lymphoma (NHL).[3] The single most important risk factor for developing PTLD is EBV infection. This association is well established and 80–90% of PTLD cases are associated with primary EBV infection or reactivation of previously acquired EBV.[4]

Most cases of PTLD occur as nodal diseases, but they can present with localized symptoms involving organs such as the gastrointestinal tract, lungs, skin, liver, central nervous system, and infiltrative lesions in the allograft. Extranodal involvement occurs in more than 50% of the cases. The gastrointestinal tract is predominantly involved with an increased propensity for ulceration and perforation.[5] In our patient, the points of interest were: a) presentation as perforation, b) the neoplastic growth resected turning out as a diffuse B cell variant of nonHodgkin's lymphoma with positive Epstein barr viral serology.
